# Feature related multi-view nonnegative matrix factorization for identifying conserved functional modules in multiple biological networks

**DOI:** 10.1186/s12859-018-2434-5

**Published:** 2018-10-29

**Authors:** Peizhuo Wang, Lin Gao, Yuxuan Hu, Feng Li

**Affiliations:** School of Computer Science and Technology, Xidian University, Xi’an, 710071 China

**Keywords:** Features, Multiple biological networks, Conserved modules, Matrix factorization

## Abstract

**Background:**

Comprehensive analyzing multi-omics biological data in different conditions is important for understanding biological mechanism in system level. Multiple or multi-layer network model gives us a new insight into simultaneously analyzing these data, for instance, to identify conserved functional modules in multiple biological networks. However, because of the larger scale and more complicated structure of multiple networks than single network, how to accurate and efficient detect conserved functional biological modules remains a significant challenge.

**Results:**

Here, we propose an efficient method, named ConMod, to discover conserved functional modules in multiple biological networks. We introduce two features to characterize multiple networks, thus all networks are compressed into two feature matrices. The module detection is only performed in the feature matrices by using multi-view non-negative matrix factorization (NMF), which is independent of the number of input networks. Experimental results on both synthetic and real biological networks demonstrate that our method is promising in identifying conserved modules in multiple networks since it improves the accuracy and efficiency comparing with state-of-the-art methods. Furthermore, applying ConMod to co-expression networks of different cancers, we find cancer shared gene modules, the majority of which have significantly functional implications, such as ribosome biogenesis and immune response. In addition, analyzing on brain tissue-specific protein interaction networks, we detect conserved modules related to nervous system development, mRNA processing, etc.

**Conclusions:**

ConMod facilitates finding conserved modules in any number of networks with a low time and space complexity, thereby serve as a valuable tool for inference shared traits and biological functions of multiple biological system.

**Electronic supplementary material:**

The online version of this article (10.1186/s12859-018-2434-5) contains supplementary material, which is available to authorized users.

## Background

Recent high-throughput experimental techniques brought a large number of multi-omics data (e.g., DNA sequence data, mRNA, miRNA, methylation, copy number variation, etc.) in different conditions (e.g., tissue types and disease states). Comprehensive analysis of these multiple biological data is non-trivial for more profound understanding of the whole biological system [1]. As a promising tool for integrative analyzing large-scale biological data, network-based approach is successful in discovering biological meaning patterns. However, most of the network-based works only concern single biological data that is insufficient to simultaneously analyze multi-omics or multiple conditions data and hinder us from capturing comprehensive information on total system. In order to settle this issue, more complex models, namely multiple networks or multi-layer network models [2, 3], have been introduced. The multiple networks, which can be created by incorporating multiple types of connection and constituting the environment to describe systems interconnected through different categories of connections, bring us a new insight into biological mechanism and medicine research in a comprehensive level [4, 5].

One significant task in multiple biological networks is to detect conserved functional modules, for the reason that the biological networks across different type of tissues, cancers or disease states have many shared patterns or underlying common cellular functional organizations, which can be represented as module structures. For example, cancers of disparate organs have many shared features [6], including rapid cell proliferation, the ability to migrate and avoiding immune destruction, etc. [7]. Understanding these common traits by identifying the underlying conserved function modules are key to gaining insight into cancer physiology and ultimately to prevent cancer. Moreover, as another example, identifying common features in biological networks across distant species can reveal evolvement relations and fundamental principles [8, 9].

Despite the great importance of extracting conserved modules in multiple biological networks, it is highly difficult to develop an effective and efficient algorithm because of two reasons. First, it is hard to characterize features of conserved modules due to the more complicated structure of multiple networks. Second, multiple networks pose a great challenge for designing efficient algorithms, since multiple networks have larger scale than single network and how to reduce time and space complexity is need to address. To handle these issues, a simple strategy is to summarize a collection of heterogeneous data into a single integrated network and use graph-based clustering on it. However, this strategy can bring about the substantial information loss. Recent years, researches developed methods on module discovery in multiple networks, such as a heuristic algorithm to mine frequent coherent dense subgraphs on unweighted networks [10], tensor based optimization algorithm [11], generalized singular decomposition based method [12], and modularity based optimization algorithm [9]. However, these methods are either limited to cluster on unweighted networks [10] or take a lot of time and memory for running [9, 11, 12]. Almost at the same time, the multi-view clustering approaches from machine learning field were also put forward to cluster for integrated data [13–16]. In these approaches, each data object is comprised of different representations (views) that provide compatible and complementary information for better clustering. However, most of these multi-view clustering methods assume that all views consist of the same set of data objects, which is not suitable to some circumstance. Moreover, these methods always separately analyze the structure of each network and concatenate the results, which greatly increase the dimensionality of the space.

In this paper, we develop an approach, called ConMod, to discover *Con*served functional *Mod*ules in multiple biological networks. Instead of mining each biological network individually, ConMod describes the networks as two feature matrices and performs a multi-view clustering approach based on non-negative matrix factorization (NMF) in these two matrices only. Our main contributions of the proposed approach are summarized as follows:

- We introduce two features to measure the strength and distribution of each edges in multiple networks. Thus, all of the multiple networks are compressed into two feature matrices, which is the basis of detecting conserved module with a low time and space complexity.
- We adopt a multi-view symmetric NMF model based on our proposed feature matrices, which help us find consensus factors with effectiveness and efficiency.
- Our method can discover conserved modules without denoting the number of networks that a module appears. If the overall signal in the consensus factors is detected, a conserved module will be found. The results show that our method can accurate find modules that appear in more than half of all networks.

To show substantial improvements over the state-of-the-art methods, we demonstrate ConMod’s accuracy and efficiency to discover conserved modules from multiple networks in two types of synthetic datasets. Moreover, to verify the biological meaning of conserved modules, we apply ConMod in two distinct biological multiple networks: (1) 33 cancer type-specific gene co-expression networks and (2) 15 brain-specific protein interaction networks. Both two tasks demonstrate the potential to effectively identify conserved modules with significantly functional implications, such as DNA replication, ribosomal protein biosynthesis and immune response in 33 cancers’ co-expression networks and nervous system development in 15 brain PPI networks, respectively. ConMod can be used to simultaneously analyze any number of networks and straightforwardly applied to other types of networks in addition to biology.

## Methods

### Overview

The multiple networks, or multi-layer network, with *M* layers can be represented by the set $\mathcal{G}=\left\{{G}^{(1)},{G}^{(2)},\dots, {G}^{(M)}\right\}$, whose element *G*^(*t*)^ = (*V*^(*t*)^, *E*^(*t*)^, *W*^(*t*)^)(*t* = 1, 2, …, *M*) is an undirected network under consideration with vertex set *V*^(*t*)^ and edge set *E*^(*t*)^, where *N*_*t*_ = |*V*^(*t*)^| denotes the number of nodes in the network layer *t*. *G*^(*t*)^ is represented by an *N*_*t*_ × *N*_*t*_ adjacency matrix **W**^(*t*)^, where each element ${w}_{ij}^{(t)}$ is the weight of the edge between nodes *i* and *j* in the network layer *t*. *N* = |∪_*t*_*V*^(*t*)^| is the total number of nodes in multiple networks.

**Fig. 1:**
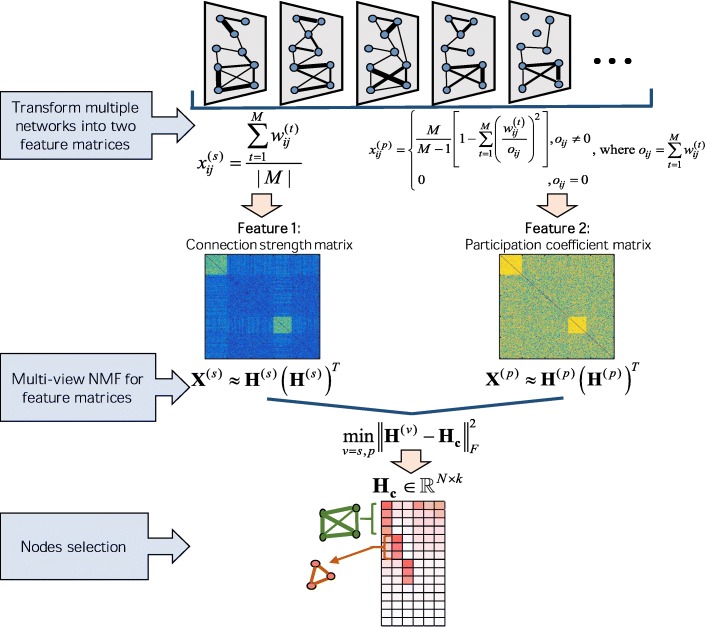
Illustration of the ConMod approach. ConMod mainly contains three steps: (1) Transforming multiple networks into two feature matrices, (2) jointly factorizing the two feature matrices into consensus factors by using multi-view NMF, (3) a soft node selection procedure from the consensus factors

The goal of our method ConMod is to identify the conserved functional modules, which exist in as many of the biological networks as possible. Figure 1 shows the flowchart of our method for detecting conserved functional modules. The basic framework of ConMod involves three steps. First, we transform multiple networks into two feature matrices, the connection strength matrix and the participation coefficient matrix, which respectively describes the overall edge weight and the degree of participation of each edge in multiple networks. Second, we jointly factorize the two feature matrices into consensus factors by using multi-view NMF. Finally, we adopt a soft node selection procedure from the consensus factors to assign the module members and then we refine the candidate modules for obtaining more accurate results. We implemented ConMod in MATLAB R2015a as a user-friendly package (https://github.com/WPZgithub/ConMod).

### Transforming multiple networks into two feature matrices

For multiple networks, conserved modules not only have densely topological structure in each network, but also broadly distribute in most networks. Based on this point, we propose two features to describe a conserved functional module. The first, connection strength, is used for characterizing whether a pair of nodes connect closely in multiple networks. The second, participation coefficient, is used for describing whether an edge is uniformly distributed across all networks. In this way, the conserved modules detection is equivalent to find node sets that consist of the edges with high connection strength and participation coefficient.

The connection strength of an edge between nodes *i* and *j*, denoted as ${x}_{ij}^{(s)}$, is defined as the average weight over all networks:

1 $$ {x}_{ij}^{(s)}=\frac{\sum \limits_{t=1}^M{w}_{ij}^{(t)}}{M}. $$

In addition, we define the participation coefficient of an edge, denoted as ${x}_{ij}^{(p)}$, as following:

2 $$ {x}_{ij}^{(p)}=\left\{\begin{array}{c}\frac{M}{M-1}\left[1-\sum \limits_{t=1}^M{\left(\frac{w_{ij}^{(t)}}{o_{ij}}\right)}^2\right],{o}_{ij}\ne 0\\ {}0\begin{array}{ccc}& & \end{array}\kern1.00em \begin{array}{cc}& \end{array}\kern0.5em ,{o}_{ij}=0\end{array},\right. $$

where ${o}_{ij}={\sum}_t{w}_{ij}^{(t)}$. The definition of the participation coefficient is first introduced by Guimera and Amaral [17, 18] to quantify the participation of a node to the different communities of a network. In our paper, we change it to measure edges and adapt it to multiple networks. Here, the participation coefficient measures whether an edge uniformly distributed among the *M* networks. The larger the value of the coefficient ${x}_{ij}^{(p)}$ is, the more uniformly distributed the edge will be in the multiple networks.

Both values of the connection strength and the participation coefficient are in [0, 1]. These two features can be used for both weighted and unweighted networks. However, for weighted networks, direct calculation of the participation coefficient for each weighted edge may not be appropriate, since the huge quantity of weakly connected edges may have very high value of participation coefficient. For example, if ${w}_{ij}^{(t)}=0.01$ for all *t* = 1, 2, ⋯, *M*, the participation coefficient ${x}_{ij}^{(p)}=1$, but the edges between nodes *i* and *j* are most likely to be neglected for module discovery due to the very low edge weight. Even though the connection strength ${x}_{ij}^{(s)}$ is small enough, the high value of participation coefficient will increase noise for conserved module detection. To handle this issue, we take the logistic transform of the input data and neglect the edges with low transformed values. Specifically, for weighted networks, the original adjacency matrix of each network is first transformed using a logistic function *L*(*w*_*ij*_) = 1/(1 + exp(*cw*_*ij*_ + *d*)), such that for *w*_*ij*_ ∈ [0, 0.3], *L*(*w*_*ij*_) ≈ 0, and for *w*_*ij*_ ∈ [0.6, 1],*L*(*w*_*ij*_) ≈ 1. This implies that *L*(0) needs to be close to 0. So we first normalize the adjacent matrix such that each element of the matrix is in [0, 1] and then we set *L*(0) = 0.0001, from which we obtain *d* = log(9999) and *c* =  − 2 log(9999).

### Computing consensus factors using multi-view symmetric NMF based on feature matrices

From now on, the relationships among *N* nodes are represented by 2-view representations, **X**^(*s*)^ and **X**^(*p*)^. Now we cluster across the two views simultaneously to find a common latent structure. Among the multi-view clustering algorithms, NMF based methods [14, 19, 20] have demonstrated strong vitality and efficiency. Based on the two feature matrices we use a multi-view NMF model [14] to find a common coefficient (or basis) matrix. Here, the original multi-view NMF model is adjusted for handling our symmetric feature matrices. Thus, we have the following objective function of the multi-view symmetric NMF:

3 $$ {\displaystyle \begin{array}{c}\underset{{\mathbf{H}}^{(v)},{\mathbf{H}}_{\mathbf{c}}}{\min}\mathrm{\mathcal{F}}=\left(\sum \limits_{v=s,p}{\left\Vert {\mathbf{X}}^{(v)}-{\mathbf{H}}^{(v)}{\left({\mathbf{H}}^{(v)}\right)}^T\right\Vert}_F^2+\sum \limits_{v=s,p}{\lambda}_v{\left\Vert {\mathbf{H}}^{(v)}-{\mathbf{H}}_{\mathbf{c}}\right\Vert}_F^2\right)\\ {}\mathrm{s}.\mathrm{t}.\kern0.5em {\mathbf{H}}^{(v)}\ge 0,{\mathbf{H}}_{\mathbf{c}}\ge 0\begin{array}{cc}\begin{array}{cccc}\begin{array}{cccc}\begin{array}{cc}\begin{array}{ccc}& & \end{array}& \end{array}& & & \end{array}& & & \end{array}& \end{array}\kern0.5em \end{array}}, $$

where ‖·‖_*F*_ denotes Frobenius norm and *λ*_*v*_ is the parameter to balance the relative weight of different views. The multi-view symmetric NMF factorize each view of symmetric data matrix **X**^(*v*)^ to a low-rank matrix representation **H**^(*v*)^, which are close to the consensus matrix **H**_**c**_.

To solve this optimization problem, we use the multiplicative update rule to minimize the objection function. Specifically, given a desired rank *k*, the algorithm iterates the following two steps until convergence. First, we fix **H**_**c**_ and minimize objective function over **H**^(*v*)^ for each view *v*. **H**^(*v*)^ is updated at each step by:

4 $$ {\left({\mathbf{H}}^{(v)}\right)}_{ik}\leftarrow {\left({\mathbf{H}}^{(v)}\right)}_{ik}\frac{{\left(2{\mathbf{X}}^{(v)}{\mathbf{H}}^{(v)}+{\lambda}_v{\mathbf{H}}_{\mathbf{c}}\right)}_{ik}}{{\left(2{\mathbf{H}}^{(v)}{\left({\mathbf{H}}^{(v)}\right)}^T{\mathbf{H}}^{(v)}+{\lambda}_v{\mathbf{H}}^{(v)}\right)}_{ik}},v=s,p. $$

Second, fixing **H**^(*v*)^ for each *v*, we take the derivative of the objective ℱ over **H**_**c**_ and obtain an exact solution:

5 $$ {\mathbf{H}}_{\mathbf{c}}=\frac{\sum \limits_{v=s,p}{\lambda}_v{\mathbf{H}}^{(v)}}{\sum \limits_{v=s,p}{\lambda}_v}\ge 0. $$

Since the objective function is non-convex, one should perform many repetitions and choose the minimizer of the objective function as the final solution.

### Selecting nodes from the consensus factors

Once the consensus matrix **H**_**c**_ is obtained, the cluster label of data point *i* could be computed as argmax_*k*_(**H**_**c**_)_*i*, *k*_. However, it will be meaningless to use this hard clustering process in most biological networks. In gene networks, for instance, some genes are multifunctional, such as the broadly expressed transcription factors and the crosstalk of gene pathways. Besides, some genes are inactive in any module in some specific conditions. Therefore, we adopt a soft node selection procedure to obtain modules with biological meaning. The nodes are selected if they have relatively large absolute values of the weighted factors **H**_**c**_. Specifically, we calculated the *z*-score for each column of **H**_**c**_ by.

6 $$ {z}_{ij}=\frac{{\left({\mathbf{H}}_{\mathbf{c}}\right)}_{ij}-{\mu}_{{\left({\mathbf{H}}_{\mathbf{c}}\right)}_{\cdotp j}}}{\sigma_{{\left({\mathbf{H}}_{\mathbf{c}}\right)}_{\cdotp j}}}, $$

where ${\mu}_{{\left({\mathbf{H}}_{\mathbf{c}}\right)}_{\cdotp j}}=\frac{1}{N}\sum {\left({\mathbf{H}}_{\mathbf{c}}\right)}_{ij}$ and ${\sigma}_{{\left({\mathbf{H}}_{\mathbf{c}}\right)}_{\cdotp j}}^2=\frac{1}{N-1}\sum {\left({\left({\mathbf{H}}_{\mathbf{c}}\right)}_{ij}-{\mu}_{{\left({\mathbf{H}}_{\mathbf{c}}\right)}_{\cdotp j}}\right)}^2$. We assign node *i* as a member of a module, if *z*_*ij*_ > *θ*. The threshold *θ* is typically in [2, 5] for most cases such that the selected nodes have significant signals in the consensus factors.

Finally, two modules with $\frac{\left|{C}_x\cap {C}_y\right|}{\min \left\{\left|{C}_x\right|,\left|{C}_y\right|\right\}}>0.5$ are merged and the modules whose sizes are smaller than five are removed, where *C*_*x*_ is the members set of module *x*.

### Complexity analysis

We first discuss the time complexity of our method. If the input networks are in the form of full matrix, the time complexity of computing two feature matrices is constant. While if the input networks are in the form of sparse matrix, its time complexity is *O*(*Me*), where *e* is the average number of edges of each network. Moreover, the time cost of the multi-view NMF procedure is *O*(*lkN*^2^), where *l* is the number of iterations. The time complexity of selecting nodes from consensus factors is *O*(*kN*). Therefore, the overall time cost is *O*(*Me*) + *O*(*lkN*^2^) + *O*(*kN*). Since *N* − 1 ≤ *e* ≤ *N*(*N* − 1)/2, then the total time complexity of ConMod is *O*((*lk* + *M*/2)*N*^2^) in the worst case and *O*(*lkN*^2^) in the best case, demonstrating the efficiency of our method.

Then we discuss the space complexity. Multiple networks $\mathcal{G}=\left\{{G}^{(1)},{G}^{(2)},\dots, {G}^{(M)}\right\}$ requires space *O*(*N*^2^*M*). However, our method compress the multiple networks into two feature matrices, and use multi-view NMF for conserved module detection, whose space complexity is *O*(2*N*^2^) and *O*(2*Nk*), respectively. Thus, the overall space complexity of ConMod is *O*(2*N*^2^), which has nothing to do with the number of networks, demonstrating the efficiency of our approach on space complexity.

### Module validation

We use a permutation test to assess the significance of functional modules across multiple networks. This allows identifying the specific conditions where each module is detected. Here, we use the cluster quality [12] as a measurement to calculated a *p*-value indicating the significance of one module in each network. The cluster quality is defined as:

7 $$ {q}_t=\frac{\mathrm{the}\ \mathrm{density}\ \mathrm{within}\ \mathrm{the}\ \mathrm{module}\ \mathrm{in}\ {G}^{(t)}}{\mathrm{the}\ \mathrm{density}\ \mathrm{outside}\ \mathrm{the}\ \mathrm{module}\ \mathrm{in}\ {G}^{(t)}}. $$

The *p*-value is computed as the proportion of the random modules with the cluster quality larger than *q*_*t*_. Raw *p*-values are corrected by using the method of Benjamin-Hochberg [21] and the corrected *p*-values below 0.01 are regarded as significant existing of a module in a specific network.

## Results and discussion

In this section, we first present simulation studies to demonstrate the performance of ConMod to detect conserved modules in synthetic multiple networks. We compare ConMod with four state-of-the-art methods, including NetsTensor [11], SC-ML [16], multi-view pairwise co-regularized spectral clustering (pairwiseCRSC) [15] and multi-view centroid-based co-regularized spectral clustering (centroidCRSC) [15]. NetsTensor introduced a tensor-based computational framework to identify recurrent heavy subgraphs in multiple biological networks. SC-ML modeled each graph layer as a subspace on a Grassmann manifold and then efficiently merge these subspaces find a unified clustering of the vertices. PairwiseCRSC and centroidCRSC employed a spectral clustering-based co-regularization framework for clustering across multiple views. Furthermore, to test whether ConMod is effective for finding conserved modules with meaningful biological functions, we apply ConMod to two sets of real biological networks, a set of 33 cancer type-specific gene co-expression networks and a set of human 15 brain tissue-specific protein interaction networks.

### Results on synthetic networks

#### Simulation data

To test the performance, we first evaluate our method using synthetic networks. We generate two sets of synthetic networks that contain different types of conserved modules: (1) conserved modules are common to a given set of networks and (2) conserved modules are present only in a subset of networks and they are the overlapping parts of specific modules across different networks.

**Fig. 2:**
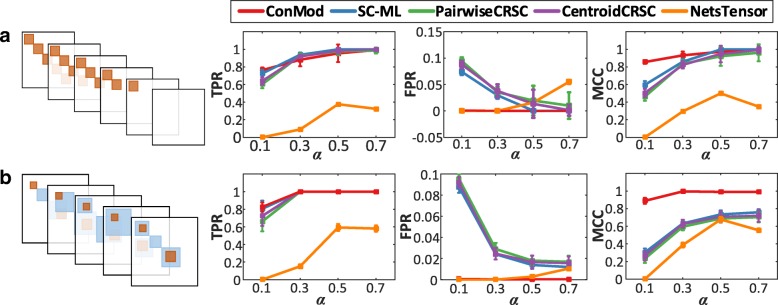
Performance in terms of TPR, FPR and MCC with different *α*. **a** The conserved modules are common to a given set of networks. **b** The conserved modules are the overlapping parts of specific modules across different networks

We consider the first type of synthetic multiple networks with *M*=30 networks and *N*=500 nodes. We generate five modules with 80 nodes in each module and these modules are randomly assigned into 25, 20, 15, 10 and 5 networks, respectively. In this way, each network contains up to five modules. In each network, we connect nodes with a possibility of *α* (0 < *α* < 1) inside each module and the nodes belonging to different modules are connected with a possibility of *β* (0 < *β* < *α*). An example is shown in Fig. 2a. In order to introduce edge weights, we embed Gaussian noise on the networks (See more details in Additional file 1).

For the second type of synthetic dataset, we consider multiple networks with *M*=15 networks and *N*=500 nodes. In each network, a module consists of two parts, a common part, in which the nodes are common to a set of networks, and a specific part, in which nodes present only in its individual network. The common parts of every module are regarded as conserved modules in this case. We set two conserved modules of this type for this synthetic dataset. A conserved module has 50 nodes and another has 40 nodes. An example is shown in Fig. 2b. Other procedures for synthetic networks construction is the same as mentioned above (See more details in Additional file 1).

In this study, we experiment on synthetic networks with *α* = 0.1, 0.3, 0.5 and 0.7 and *β* = 0.05. Lower value of *α* means modules are fuzzier and harder to detect.

#### Evaluation measures

We use true positive rate (TPR), false positive rate (FPR) and Matthew’s correlation coefficient (MCC) [22] to quantify the performance of methods, which are defined as:

8 $$ TPR=\frac{TP}{TP+ FN} $$

9 $$ FPR=\frac{FP}{FP+ TN} $$

10 $$ MCC=\frac{TP\times TN- FP\times FN}{\sqrt{\left( TP+ FP\right)\left( TP+ FN\right)\left( TN+ FP\right)\left( TN+ FN\right)}}, $$

where TP is the number of true positives, TN is the number of true negatives, FP is the number of false positives and FN is the number of false negatives. A TP decision assigns two related nodes to the same module. A TN decision assigns two unrelated nodes to different modules. An FP decision assigns two unrelated nodes to the same module. An FN decision assigns two related nodes to different modules. MCC returns a value in [−1, 1]. A value of + 1 represents a perfect prediction, 0 is no better than random prediction and − 1 indicates total disagreement between prediction and observation.

#### Performance

We generate synthetic datasets with different value of *α*. For our method, we use the parameters *λ*_*s*_ = 0.01, *λ*_*p*_ = 0.05 and *θ* = 2. The effects of parameters will be discussed later in more detail. All experiments are repeated 50 times on random generated datasets and the average results are reported for consistency. Figure 2 shows the examples of synthetic multiple networks with different type of conserved modules and the accuracy of each method in terms of TPR, FPR and MCC. ConMod outperforms the other methods in various value of *α* whenever the conserved modules are common to a given set of networks (Fig. 2a) or are the overlapping parts of specific modules across different networks (Fig. 2b). In particular, ConMod performs the best when the module structures are fuzzier (*α* = 0.1).

**Fig. 3:**
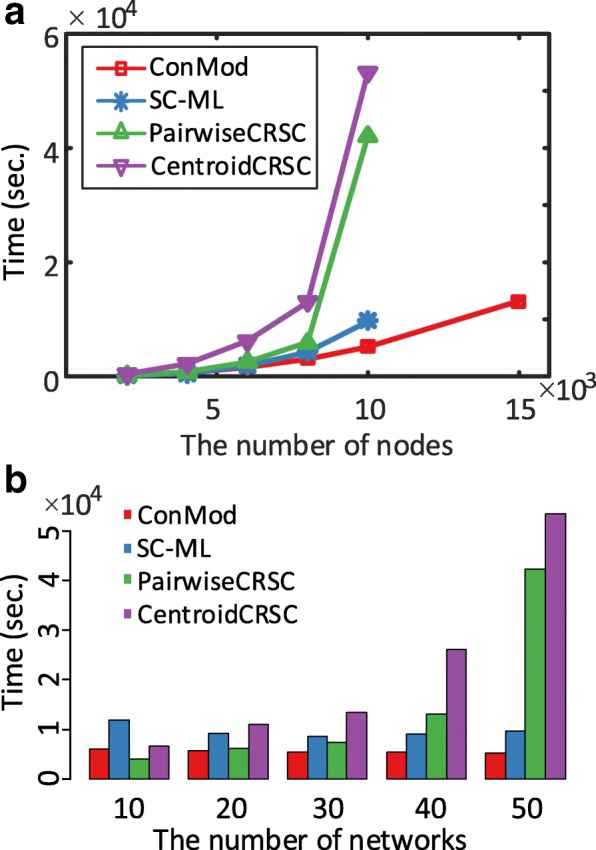
Running time evaluation. **a** The running time when varying the number of nodes and keeping the number of networks as 50. **b** The running time when varying the number of networks and keeping each network size as 10,000

Next, we evaluate the efficiency of ConMod. We conduct the experiments on a 2.10GHz desktop with 128GB memory. Figure 3a shows the running time when varying the number of nodes and keeping the number of networks as 50. Figure 3b shows the running time when varying the number of networks and keeping each network size as 10,000. We do not compare with NetsTensor and omit the results of SC-ML, PairwiseCSRC and CentroidCSRC when the number of nodes is larger than 10,000 because of their high memory and running time cost. As can be seen from Fig. 3, the running time of ConMod is very low and is almost not affected by the number of networks, especially in large scale multiple networks. Additional figures regarding the other number of networks and nodes are put in the additional file (Additional file 1: Figure S1 and S2).

### Conserved functional modules in cancer type-specific gene co-expression networks

In this section, we apply ConMod to multiple large-scale gene co-expression networks of 33 cancers. We aim at finding common signatures and biological functions in different cancers by identifying conserved functional modules. Such conserved gene co-expression modules can help reveal the gene expression regulatory basis for common traits in cancer [23].

We download the mRNA-sequencing data of all available 33 cancer types from The Cancer Genome Atlas (TCGA) database (https://portal.gdc.cancer.gov/). For each cancer type, we only select samples labeled as tumor. The Fragments Per Kilobase Million (FPKM) of each gene is transformed by log_2_(FPKM + 1). For each cancer type, coding genes with FPKM > 1 in more than 50% of all samples are selected. Then the intersection of expressed genes in all cancer types are used for constructing cancer type-specific gene co-expression networks based on Pearson’s correlation coefficient. Meaningful relations are selected based on first-order partial correlation and information theory by PCIT R package [24]. Finally, we obtain a set of 33 cancer type-specific gene co-expression networks with 7,526 genes for each network.

We compare the performance of ConMod with NetsTensor [11], SC-ML [16], pairwiseCRSC [15] and centroidCRSC [15] by assessing the biological relevance of identified conserved functional modules. Here, we perform systematic enrichment analysis for genes of each module using Gene Ontology (GO) biological process [25, 26]. We use precision, recall and f-score as the evaluation measures in this case. Precision is defined as the fraction of predicted modules that significantly overlap with reference gene sets. Recall is defined as the fraction of reference gene sets that significantly overlaps with predicted modules. F-score is defined as the harmonic mean of precision and recall. We calculate statistical significance *p*-value using Fisher’s exact test and raw *p*-values were corrected using the method of Benjamin-Hochberg [21].

**Fig. 4:**
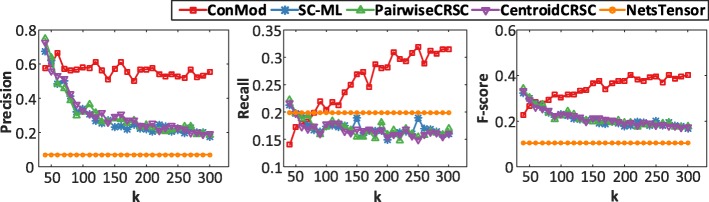
Precision, recall and f-score with different *k* in 33 cancer type-specific gene co-expression networks. Biological relevance of conserved modules is evaluated by GO biological process

Figure 4 shows the performance of ConMod and other methods in terms of precision, recall and f-score w.r.t. different number of candidate modules *k*. We clearly see that ConMod is more stable than other methods and performs the best in most cases. Besides, we can see that the f-score is high enough when *k*=150 and has no significant increase after *k*=150, which can provide a reference for the selection of *k*. Note that NetsTensor does not need to specify the number of modules in advance, however it does not perform well because of the low node coverage and high overlap between discovered modules.

**Fig. 5:**
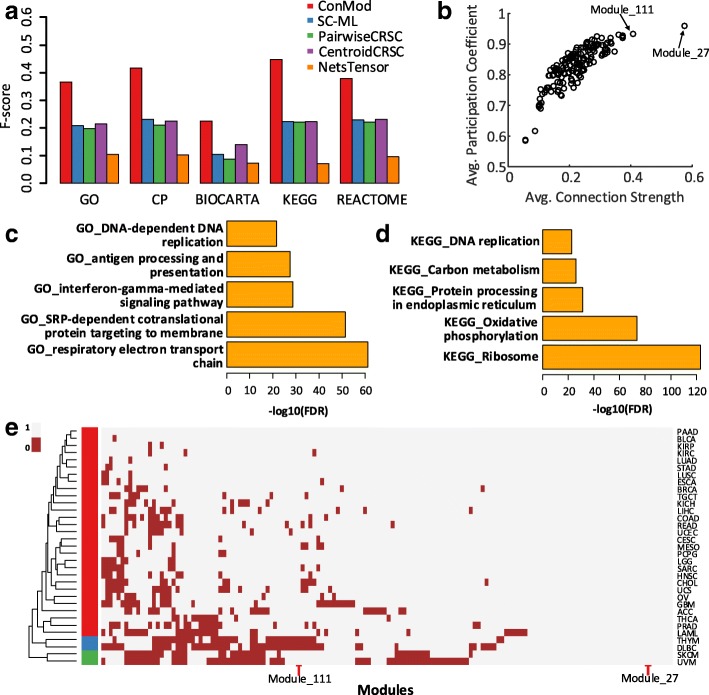
Illustration of results on a set of 33 cancer type-specific gene co-expression networks. **a** F-score of five methods. Gene modules found by each method are evaluated by multiple gold-standard gene set annotations. **b** The scatter plot for the average value of connection strength and participation coefficient of each module. Each point represents an identified module. **c** Top five significant biological process enriched by the identified modules. **d** Top five significant KEGG pathways enriched by the identified modules. **e** Heat map of distribution of modules in multiple cancers. Each row represents a conserved module and each column corresponds to a cancer type. If a module significantly distributes in a cancer’s network (BH-adjusted *p*-value< 0.01, permutation test), it will be labeled as 1. Otherwise, a module will be labeled as 0

Next, after parameter optimizations, we set *k*=150 and *θ* = 3.5 and obtain 150 conserved functional modules covering 7,182 genes. The average module size is 113.2. We evaluated the resulting gene modules using multiple gold-standard gene set annotations from MsigDB [27] of GSEA [28], including the biological process category of Gene Ontology (GO) [25, 26], Canonical pathways (CP), Biocarta [29], KEGG [30] and REACTOME [31]. ConMod achieves higher f-scores than other four methods using all reference sets (Fig. 5a). We find that 86 (57%) and 60 (40%) of conserved modules are significantly enriched in at least one GO biological process and KEGG pathway (BH-adjusted *p*-value< 0.05). We present the top five significant GO biological processes and KEGG pathways in Fig. 5c and d respectively. We observe that these biological functions are related to ribosome protein, energy metabolism, cell cycle and immune response. Most of these functions are necessary to maintain a cell’s life. These modules, acting as housekeeping roles, universally expressed in different tissues. However, cancers require a great deal of DNA replication and protein synthesis. Thus, most of the conserved modules and their functions are also closely associated with cancer. In particular, two significant GO biological processes, antigen processing and presentation and interferon-gamma-mediated signaling pathway, are both essential for immune response, which is often observed to be inhibited in the tumor microenvironment [7, 32]. In addition, we test the relationship between the functional modules and cancer driver genes [33, 34]. By following a previous work [35], we utilized 2,372 genes from the Network of Cancer Genes (NCG) [36] as benchmarking cancer genes, including 711 known cancer genes from the Cancer Gene Census (CGC) [37]. We use Fisher’s exact test to validate whether the modules are significantly associated with benchmark cancer genes (BH-adjusted *p*-value< 0.05) and find that our method can get more modules with significantly enriched cancer driver genes than other methods (Additional file 1: Figure S3). This result indicates that the conserved functional modules identified by our method are able to reveal the characteristics of cancer.

We compute the average value of connection strength and participation coefficient respectively for each conserved module, and we observe that the two features are highly correlated (Pearson correlation coefficient *r*=0.83) (Fig. 5b). It is easily understood that a dense module conserved in more networks tend to has larger connection strength. After module validation, we can know how the conserved modules distribute in multiple networks (Fig. 5e). We consider that a module exists in a network if its Benjamin-Hochberg adjusted *p*-value< 0.01 using a permutation test. Modules that do not exist in more than half of all networks are removed. From Fig. 5e we observe that about 25% of identified modules are common in all cancers and almost all modules are conserved in more than half of these cancers. Furthermore, similar cancers can be naturally clustered together only based on the distribution of identified modules (see the hierarchical clustering for cancers in Fig. 5e), such as SKCM (Skin Cutaneous Melanoma) and UVM (Uveal Melanoma); and THYM (Thymoma) and DLBC (Lymphoid Neoplasm Diffuse Large B-cell Lymphoma). Actually, SKCM and UVM are two types of melanoma, THYM and DLBC are both originated in the lymphatic system that participates in immune response.

**Fig. 6:**
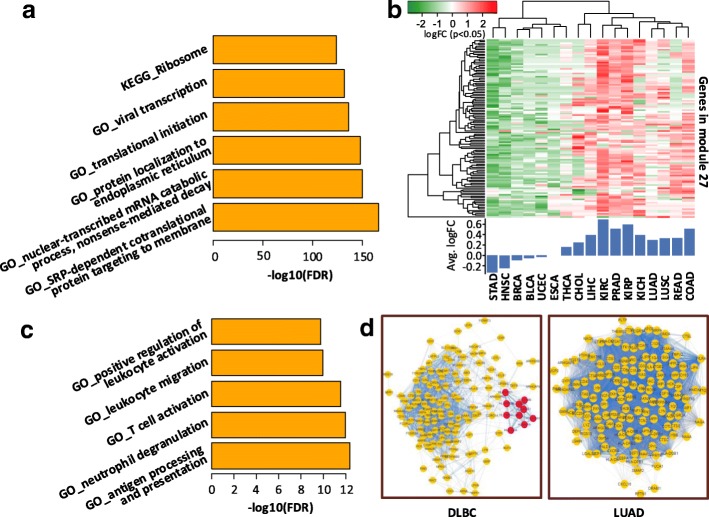
Illustration of module 27 and module 111. **a** Top significant biological terms enriched by the genes of module 27. The top five GO terms in biological process and the only one enriched KEGG pathway are displayed. **b** The heat map illustrates the log2 fold-change of gene expression in module 27 for each cancer relative to normal samples. The bar plot under the heat map shows the average log2 fold-change of all genes in module 27. Cancers with at least five normal samples are selected. **c** Top significant GO terms in biological process enriched by the genes of module 111. **d** The presentations of module 111 in DLBC and LUAD. The module mainly split into two dense sub-modules in DLBC, a small part consists of MHC class II genes and a large part consists other immune-related genes

Here, we take module 27 and module 111 as examples. Module 27, which has the largest connection strength and participation coefficient (Fig. 5b), significant exists in all cancers (BH-adjusted *p*-value < 0.01, permutation test). This module contains 112 genes, among which 77 genes encode ribosomal protein (RP). RPs, which participate in ribosome composition, is widely distributed among various tissues. Ribosomes have the functions of DNA repair, cell development regulation and cell differentiation. In addition to their essential housekeeping roles in ribosome biogenesis and protein production in all cells, RPs were reported to change in the rate of ribosome biogenesis that regulate tumorigenesis [38–40]. In order to investigate the alterations gene expression patterns of this RP related gene module in different cancers, we compute the log2 fold-change for significantly differentially expressed genes in module 27 (Fig. 6b). 17 cancers with at least five normal samples are selected for this experiment. For each cancer, we use DESeq2 [41] to detect differentially expressed genes relative to normal samples. As shown in Fig. 6b, most genes of module 27 are significantly up-regulated in more than half cancers, especially in COAD (Colon Adenocarcinoma), LIHC (Liver Hepatocellular Carcinoma), PRAD (Portal Prostate adenocarcinoma) and three kinds of kidney cancers (KIRC (Kidney renal clear cell carcinoma), KIRP (Kidney renal papillary cell carcinoma) and KICH (Kidney Chromophobe)). Even though cancer cells require continuous ribosome biogenesis and protein translation to maintain their high proliferation rate [39], it is reported that many RP genes have been found overexpressed in cancer and their mutations have been detected in the genome of cancer cells [40, 42], for example, in prostate cancer [43, 44] and in colorectal cancer [45, 46]. Hence, targeting ribosome biogenesis of tumor cells could be an effective strategy [40].

Module 111 consist of 137 genes. Genes in this module mainly involve in antigen processing and neutrophil, leukocyte or T cell related processes, which are all closely related with cancers due to their important roles in immune system (Fig. 6c). This module, however, does not exist in THYM and DLBC (Fig. 5e). Actually, the module mainly splits into two dense sub-modules in THYM and DLBC respectively, but maintains a complete module in the rest cancers, e.g. in LUAD (Lung Adenocarcinoma) (Fig. 6d). In particular, module 111 in DLBC consist of a large sub-module and a small sub-module. The small part comprise 10 genes (CD74, HLA-DQB1, HLA-DRB1, HLA-DQA1, HLA-DRB5, HLA-DMA, HLA-DRA, HLA-DPB1, HLA-DPA1, HLA-DMB), all of which are MHC (major histocompatibility complex) class II genes in HLA (human leucocyte antigen). The separation of the two sub-modules results from the weak correlation in expression between the MHC class II genes in the small sub-module and other immune-related genes in the large sub-module, suggesting a disruption of the co-operation of these genes to exert immunity responses. Actually, DLBC is a cancer of B cells. Cancerous B cells can not normally produce MHC class II molecules, which are exported to B cell’s surface and interact with their intended T cells to initiate immune response [47].

### Conserved function modules in human brain tissue-specific interaction networks

The human brain is a complex system organized by structural and functional relationships between its functional regions, such as the thalamus, brainstem and other brain tissues. Recently, multiple brain networks and their applications in neuroscience have successfully uncovered brain-associated features [48, 49]. We now aim to identify conserved protein modules across human tissue-specific networks, which may reveal important function units for brain activity.

We run ConMod on a set of 15 human brain tissue-specific protein interaction networks [50] to find conserved protein modules. There are 2,721 proteins in total. It should be noted that, different from 33 cancer type-specific networks in the above section, all networks of this dataset are unweighted and they have different number of nodes.

**Fig. 7:**
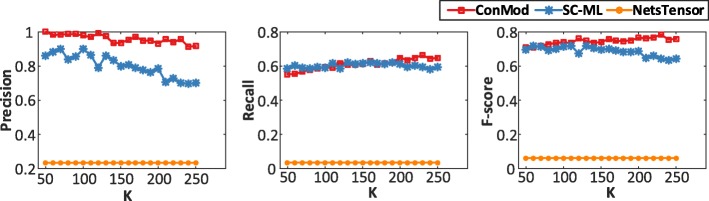
Precision, recall and f-score with different *k* in 15 brain tissue-specific protein interaction networks. Modules are evaluated by GO biological process

We compare ConMod with SC-ML and NetsTensor on this data because other methods are not suitable for the dataset in which the set of data objects is different in each network. Figure 7 shows the performance of ConMod and other methods in terms of precision, recall and f-score w.r.t. different number of candidate modules *k*. As is shown, ConMod outperforms SC-ML and NetsTensor in precision for all settings of *k* while maintaining comparable recall values. As an average, ConMod has a better performance in f-score.

**Fig. 8:**
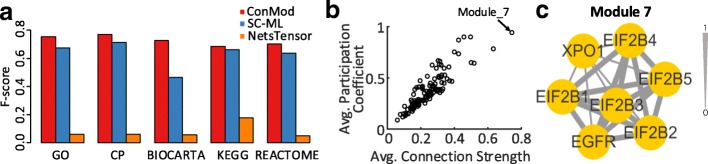
Illustration of results on 15 brain tissue-specific interaction networks. **a** F-score of three methods. Gene modules found by each method are evaluated by multiple gold-standard gene set annotations. **b** The scatter plot for the average value of connection strength and participation coefficient of each module. **c** A case of identified conserved module that are significant related with glial cell development (FDR = 3.94E-11)

After parameter optimizations, we set *k*=120 and *θ* = 4 and obtained 114 conserved functional modules covering 1,414 genes. The average module size is 23.2. We evaluated these modules using multiple gold-standard gene set annotations as the same procedure mentioned in the above section. As shown in Fig. 8a, ConMod achieves higher f-score when evaluated using all reference sets. The identified conserved modules mainly relate to nervous system development, mRNA processing, etc. (Additional file 1: Figure S4). Here, we take module 7 as an example (Fig. 8c). Module 7, which has the largest connection strength and participation coefficient in this dataset (Fig. 8b), consists of seven proteins with a significant number (6) of proteins (EIF2B1, EIF2B2, EIF2B3, EIF2B4, EIF2B5 and EGFR) for glial cell development (BH-adjusted *p*-value = 3.94E-11). Leegwater et al. [51] reported that the gene mutations of the five subunit proteins (EIF2B1, EIF2B2, EIF2B3, EIF2B4 and EIF2B5) of EIF2B complex can lead to white matter abnormalities, a serious hereditary neurodegenerative disease. Another important gene is EGFR, which is widely distributed in glial cells of mammalian brain. EGFR activation is essential for the proliferation of multipotent neural precursors, as well as the survival, migration, and differentiation of the immature daughter cells [52].

### Parameter discussion

There are four parameters in our ConMod method: the regularization parameters *λ*_*s*_ and *λ*_*p*_ in multi-view NMF, the number of modules *k* and the threshold *θ* for nodes selection. We first discuss the influence of parameters *λ*_*s*_ and *λ*_*p*_. Following a similar approach as proposed in Ref. [14], we set *λ*_*v*_ to be the same for convenience, that is *λ*_*v*_ = *λ*_*s*_ = *λ*_*p*_, and varying it from 10^−3^ to 1 on two synthetic datasets with *α* = 0.1 and *α* = 0.3. The optimal values appear when *λ*_*v*_ is around 0.01 and the accuracy is relatively stable when *λ*_*v*_ < 0.1 (Additional file 1: Figure S5). We aim at finding conserved modules, thus we let the participation coefficient has a larger effect by denoting *λ*_*s*_ = 0.01 and *λ*_*p*_ = 0.05 for all the experiments.

The selection of the parameter *k* has a significant effect on the results. While the choice of *k* is often data-dependent and is a long-standing open problem. The lower *k* of the reduced space is a key parameter for this study. For the synthetic datasets, we set *k* as the real number of modules. However, for real biological datasets that the real number of modules is unknown, we select a proper *k* by assessing the enrichment rate of gene modules with respect to GO biological process. A low *k* with a relative high f-score is selected for each datasets. We have shown in the experiments that we choose *k*=150 for multiple co-expression networks of cancers (Fig. 4; Additional file 1: Figure S6) and *k* = 120 for multiple brain-specific protein interaction networks (Fig. 7; Additional file 1: Figure S7).

The parameter *θ* determines the size of a module. A larger *θ* means a small size of module, but a more significant signal in the consensus factors. *θ* is generally larger than 2, because the corresponding *p*-value is smaller than 0.05. In our experiments we choose *θ* = 2 for all the synthetic datasets, *θ* = 3.5 for multiple co-expression networks of cancers (Additional file 1: Figure S6) and *θ* = 4 for multiple brain-specific protein interaction networks (Additional file 1: Figure S7). The reason for selection these values of parameter *θ* is that we try to keep small size of modules and a high coverage of total number of nodes while ensure a high accuracy.

## Conclusion

In this study, we present ConMod, a method for identifying conserved functional modules in multiple biological networks. Experiments on two types of simulated data show that ConMod has competitive performance in accuracy and efficiency when compared with four state-of-the-art methods. Effectiveness of ConMod on real biological networks is further demonstrated using cancer type-specific gene co-expression networks and brain tissue-specific protein interaction networks. The major advantage of our approach is that the proposed two features, connection strength and participation coefficient, give a new insight into characterizing the structure of multiple networks, which compress multiple networks without a lot of information loss. Furthermore, ConMod is very flexible for identifying conserved modules in multiple networks, because it can be applied to a set of any number of unweighted and weighted networks, and it can also be easily extended to other types of networks outside biology.

Besides the importance of conserved modules in biological networks, specific modules also have great significance for better understanding biological mechanism and even for precision medicine. Although the introduced two features are helpful for mining conserved modules, they might not fully characterize the module structures in multiple networks. Thus, it is crucial to design an algorithm to detect conserved modules and condition-specific modules simultaneously. We hope that future work on integrating more types of biological networks will provide greater insight into pathway structures and highlight network-level dynamics underlying biological responses.

## Additional file

Additional file 1: Supplement containing information on the multi-view symmetric NMF method, the construction of synthetic networks and figures about additional results. (PDF 336 kb)

## Abbreviations

BH: Benjamin-Hochberg
COAD: Colon Adenocarcinoma
CP: Canonical pathways
DLBC: Lymphoid Neoplasm Diffuse Large B-cell Lymphoma
FPKM: Fragments Per Kilobase Million
FPR: False positive rate
GO: Gene Ontology
HLA: Human leucocyte antigen
KICH: Kidney Chromophobe
KIRC: Kidney renal clear cell carcinoma
KIRP: Kidney Renal Papillary Cell Carcinoma
LIHC: Liver Hepatocellular Carcinoma
LUAD: Lung Adenocarcinoma
MCC: Matthew’s correlation coefficient
MHC: Major histocompatibility complex
NMF: Non-negative matrix factorization
PRAD: Portal Prostate Adenocarcinoma
RP: Ribosomal protein
SKCM: Skin Cutaneous Melanoma
TCGA: The Cancer Genome Atlas
THYM: Thymoma
TPR: True positive rate
UVM: Uveal Melanoma
