# Supplementary material for: Feature related multi-view nonnegative matrix factorization for identifying conserved functional modules in multiple biological networks
Source: BMC Bioinformatics. 2018 Oct 29;19:394. doi: 10.1186/s12859-018-2434-5 (PMC6206826; doi:10.1186/s12859-018-2434-5)
Supplement: Supplementary file 1 — Supplement containing information on the multi-view symmetric NMF method, the construction of synthetic networks and figures about additional results. (PDF 336 kb) [file 12859_2018_2434_MOESM1_ESM.pdf]

Supplementary Material

Feature Related Multi-view Nonnegative Matrix Factorization for Identifying Conserved Functional Modules in Multiple Biological Networks

Peizhuo Wang, Lin Gao*, Yuxuan Hu, Feng Li
School of Computer Science and Technology, Xidian University, 710071 Xi'an, China
E-mails: wangpeizhuo_37@163.com, lgao@mail.xidian.edu.cn, 772437602@qq.com, lifeng_10_28@163.com
* To whom correspondence should be addressed

Supplementary Methods

1. Multi-view symmetric NMF algorithm

The multi-view symmetric NMF problem minimize the following objective function:

$$\mathcal{F}(\mathbf{H}^{(v)},\mathbf{H_c}) = \sum_{v=s,p} \left\| \mathbf{X}^{(v)} - \mathbf{H}^{(v)}\left(\mathbf{H}^{(v)}\right)^T \right\|_F^2 + \sum_{v=s,p} \lambda_v \left\| \mathbf{H}^{(v)} - \mathbf{H_c} \right\|_F^2 \tag{1}$$

with the constraints $\mathbf{H}^{(v)} \ge 0$ and $\mathbf{H_c} \ge 0$, where $\mathbf{X}^{(v)}$ is a symmetric matrix for each $v = s, p$.

The objective function in eq. (1) is a non-convex function with respect to the entries of $\mathbf{H}^{(v)}$ and $\mathbf{H_c}$, and has multiple local minima. Therefore, it is unrealistic to design an algorithm to find the global minimum solution. Thus, we derive the multiplicative update algorithm to find a local minimum of this problem.

The computation of $\mathbf{H}^{(s)}$ and $\mathbf{H}^{(p)}$ are independent. Therefore, we use $\mathbf{X}$ and $\mathbf{H}$ to represent $\mathbf{X}^{(v)}$ and $\mathbf{H}^{(v)}$ for brevity. Based on the simple knowledge of linear algebra, the objective function $\mathcal{F}$ can be reformulated as follows:

$$\mathcal{F} = tr\left(\mathbf{XX}^T - 2\mathbf{XHH}^T + \mathbf{HH}^T\mathbf{HH}^T\right) + tr\left[\lambda_v\left(\mathbf{HH}^T - 2\mathbf{HH_c}^T + \mathbf{H_c H_c}^T\right)\right] \tag{2}$$

We first fix $\mathbf{H_c}$ and compute $\mathbf{H}^{(v)}$. Let ϕ_{ij} be the Lagrange multiplier for constraint $\mathbf{H}_{ij} \ge 0$. KTT condition. The Lagrange $\mathbb{L}$ is

$$\begin{aligned}\mathbb{L} = {} & tr(\mathbf{XX}^T) - 2tr(\mathbf{XHH}^T) + tr(\mathbf{HH}^T\mathbf{HH}^T) \\ & + \lambda_v tr(\mathbf{HH}^T) - 2\lambda_v tr(\mathbf{HH_c}^T) + \lambda_v tr(\mathbf{H_c H_c}^T) + tr(\Phi\mathbf{H}^T)\end{aligned} \tag{3}$$

where $\Phi = [\phi_{ij}]$. The partial derivatives of $\mathbb{L}$ with respect to $\mathbf{H}$ is:

$$\frac{\partial \mathbb{L}}{\partial \mathbf{H}} = -2\left(\mathbf{X} + \mathbf{X}^T\right)\mathbf{H} + 4\mathbf{H}^T\mathbf{H}\mathbf{H}^T + 2\lambda_v\mathbf{H} - 2\lambda_v\mathbf{H_c} + \Phi \quad (4)$$

Since $\mathbf{X}$ is symmetric, eq. (4) can be rewritten as:

$$\frac{\partial \mathbb{L}}{\partial \mathbf{H}} = -4\mathbf{XH} + 4\mathbf{H}^T\mathbf{H}\mathbf{H}^T + 2\lambda_v\mathbf{H} - 2\lambda_v\mathbf{H_c} + \Phi \quad (5)$$

Based on the KTT conditions $\phi_{ij}\mathbf{H}_{ij} = 0$, we get the following equations for $\mathbf{H}_{ij}$:

$$\phi_{ij}\mathbf{H}_{ij} = 2\left[2\mathbf{XH} - 2\mathbf{H}^T\mathbf{H}\mathbf{H}^T + \lambda_v(\mathbf{H} - \mathbf{H_c})\right]_{ij} H_{ij} = 0 \quad (6)$$

Then we can get the following updating rule:

$$\mathbf{H}_{ij} \leftarrow \mathbf{H}_{ij}\frac{(2\mathbf{XH} + \lambda_v\mathbf{H_c})_{ij}}{\left(2\mathbf{H}\mathbf{H}^T\mathbf{H} + \lambda_v\mathbf{H}\right)_{ij}} \quad (7)$$

Second, we fix $\mathbf{H}^{(v)}$ for each v , we take the derivative of the objective $\mathcal{F}$ over $\mathbf{H_c}$ and obtain an exact solution:

$$\mathbf{H_c} = \frac{\sum_{v=s,p}\lambda_v\mathbf{H}^{(v)}}{\sum_{v=s,p}\lambda_v} \geq 0 \quad (8)$$

2. Synthetic networks

2.1 Synthetic networks #1

Firstly, we set $M = 30$ networks and $N = 500$ nodes. Each network has the same node set. We generate each background network as matrix $\mathbf{W}^{(t)}$, where $t = 1, 2, \cdots, 30$. The (i, j) element of matrix $\mathbf{W}^{(t)}$ is defined as $w_{ij}^{(t)} = u$, where $u \sim \mathrm{Unif}(0,1)$. Then, we set five conserved modules $C = \{C_1, C_2, C_3, C_4, C_5\}$, where $C_k = \{x \mid x = 80(k-1) + j, j = 1, 2, \cdots, 80\}$. The underlying modules in each network are generated as follows:

$$w_{ij}^{(1)}, \cdots, w_{ij}^{(25)} = \begin{cases} 1, & \text{if } i, j \in C_1 \\ 0, & \text{others} \end{cases},$$

$$w_{ij}^{(1)}, \cdots, w_{ij}^{(20)} = \begin{cases} 1, & \text{if } i, j \in C_2 \\ 0, & \text{others} \end{cases},$$

$$w_{ij}^{(1)}, \cdots, w_{ij}^{(15)} = \begin{cases} 1, & \text{if } i, j \in C_3 \\ 0, & \text{others} \end{cases}, .$$

$$w_{ij}^{(1)}, \cdots, w_{ij}^{(10)} = \begin{cases} 1, & \text{if } i, j \in C_4 \\ 0, & \text{others} \end{cases},$$

$$w_{ij}^{(1)}, \cdots, w_{ij}^{(5)} = \begin{cases} 1, & \text{if } i, j \in C_5 \\ 0, & \text{others} \end{cases},$$

Besides, there is only one random module of size 80 in each of $\mathbf{W}^{(26)},\mathbf{W}^{(27)},\cdots,\mathbf{W}^{(30)}$.

For each network, we randomly flip $1-\alpha$ $(0\le\alpha\le1)$ fraction of 1 entries in each matrix to 0 and β $(0\le\beta\le\alpha)$ fraction of 0 entries to 1. To embed edge weight for each network, we set

$$w_{ij}^{(t)} := \begin{cases} \min\left(w_{ij}^{(t)}+\Delta^{(t)},0\right), & \text{if } w_{ij}^{(t)}=0 \\ \max\left(w_{ij}^{(t)}-\Delta^{(t)},1\right), & \text{if } w_{ij}^{(t)}=1 \end{cases},$$

where $\Delta_{ij}^{(t)}\sim N(0.25,0.1)$. In the end, we let $\mathbf{W}^{(t)}:=0.5\times\left(\mathbf{W}^{(t)}+\left(\mathbf{W}^{(t)}\right)^T\right)$ and $w_{ii}^{(t)}=0$.

2.2 Synthetic networks #2

Firstly, we set $M=15$ networks and $N=500$ nodes. Each network has the same node set. We generate each background network as matrix $\mathbf{W}^{(t)}$, where $t=1,2,\cdots,30$. The (i,j) element of matrix $\mathbf{W}^{(t)}$ is defined as $w_{ij}^{(t)}=u$, where $u\sim\text{Unif}(0,1)$. Then, we set two conserved modules $C=\{C_1,C_2\}$, where $C_1=\{x\mid x=1,2,\cdots,50\}$ and $C_2=\{x\mid x=401,402,\cdots,440\}$. The underlying modules in each network are generated as follows:

$$w_{ij}^{(1)},\cdots,w_{ij}^{(5)}=\begin{cases}1, & \text{if } i,j\in C_1 \text{ or } i,j\in P^{(t)} \\ 0, & \text{others}\end{cases},$$

$$w_{ij}^{(6)},\cdots,w_{ij}^{(10)}=\begin{cases}1, & \text{if } i,j\in C_1\bigcup C_2 \text{ or } i,j\in P^{(t)} \text{ or } i,j\in Q^{(t)} \\ 0, & \text{others}\end{cases},,$$

$$w_{ij}^{(10)},\cdots,w_{ij}^{(15)}=\begin{cases}1, & \text{if } i,j\in C_2 \text{ or } i,j\in Q^{(t)} \\ 0, & \text{others}\end{cases},$$

where $P^{(t)}$ is a set, which is randomly selected from $\{x\mid x=1,2,\cdots,500 \text{ and } x\notin C_1\}$, with size $10\times(11-t)(t=1,2,\cdots,10)$, and $Q^{(t)}$, which is randomly selected from $\{x\mid x=1,2,\cdots,500 \text{ and } x\notin C_2\}$, with size $5\times(t-2)(t=6,7,\cdots,15)$.

For each network, we randomly flip $1-\alpha$ $(0\le\alpha\le1)$ fraction of 1 entries in each matrix to 0 and β $(0\le\beta\le\alpha)$ fraction of 0 entries to 1. To embed edge weight for each network, we set

$$w_{ij}^{(t)} := \begin{cases} \min\left(w_{ij}^{(t)}+\Delta^{(t)},0\right), & \text{if } w_{ij}^{(t)}=0 \\ \max\left(w_{ij}^{(t)}-\Delta^{(t)},1\right), & \text{if } w_{ij}^{(t)}=1 \end{cases},$$

where $\Delta_{ij}^{(t)}\sim N(0.25,0.1)$. In the end, let $\mathbf{W}^{(t)}:=0.5\times\left(\mathbf{W}^{(t)}+\left(\mathbf{W}^{(t)}\right)^T\right)$ and $w_{ii}^{(t)}=0$.

Supplementary Figures

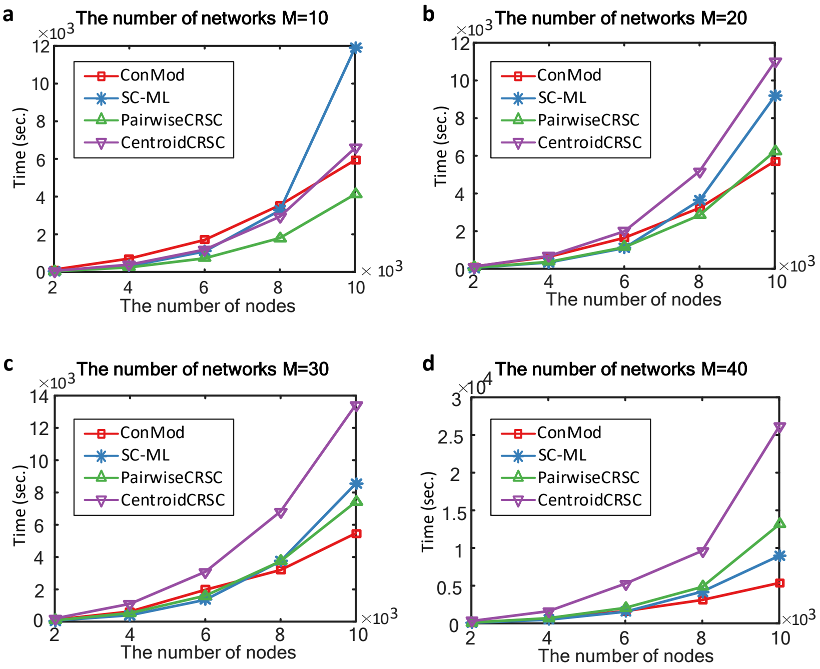

Figure S1 The running time when varying the number of nodes from 2,000 to 10,000 and keeping the number of networks as (a) 10, (b) 20, (c) 30 and (d) 40, respectively.

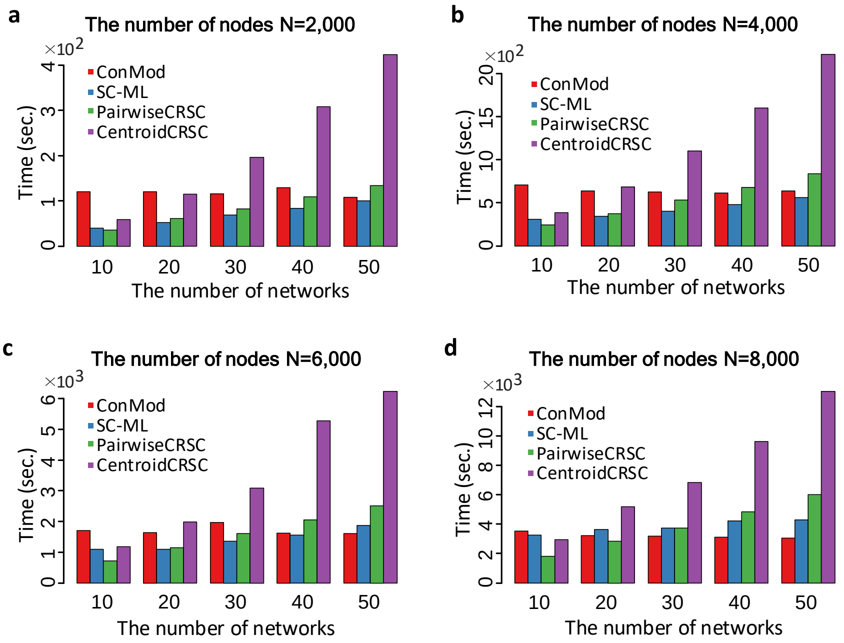

Figure S2 The running time when varying the number of networks from 10 to 50 and keeping each network size as (a) 2,000, (b) 4,000, (c) 6,000 and (d) 8,000, respectively.

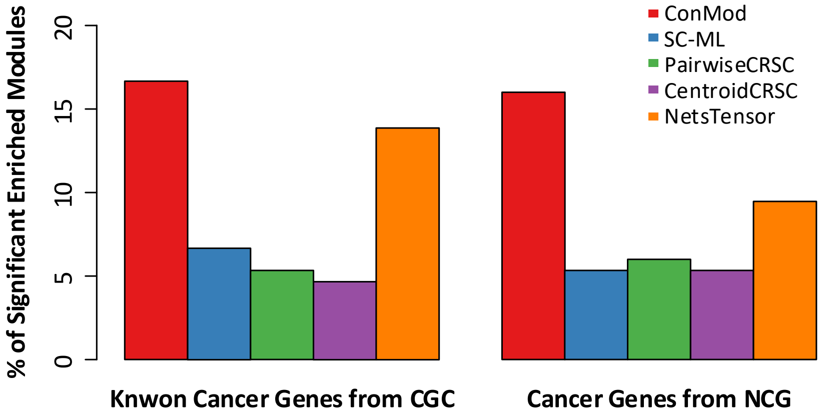

Figure S3 The percentage of modules which significant enriched into known cancer genes from CGC[1] and cancer genes from NCG[2] respectively.

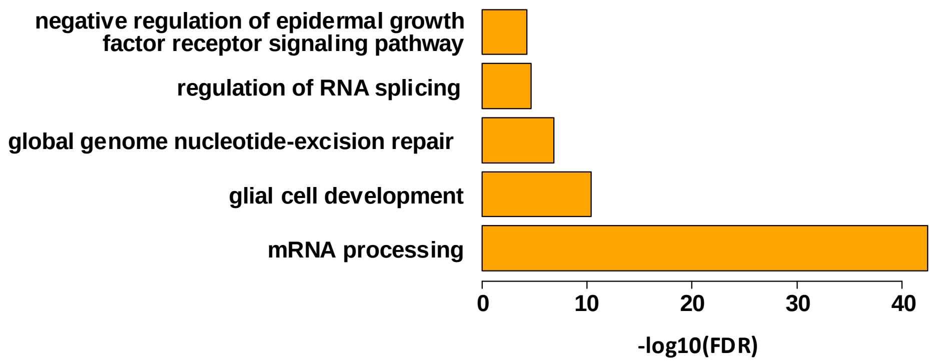

Figure S4 Significant GO biological processes of the top five conserved modules in human brain tissue-specific interaction networks.

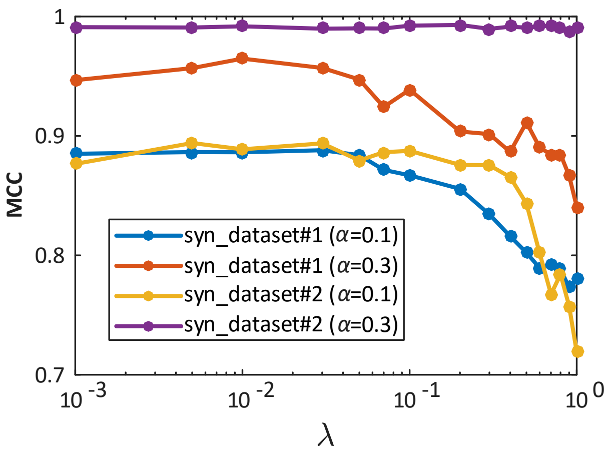

Figure S5 The average MCC on synthetic dataset #1 and #2 with $\alpha = 0.1$ and $\alpha = 0.3$ respectively when varying parameter λ_v $(\lambda_v = \lambda_s = \lambda_p)$ from .10^{-3}. to 1. The optimal values appear when λ_v is around 0.01.

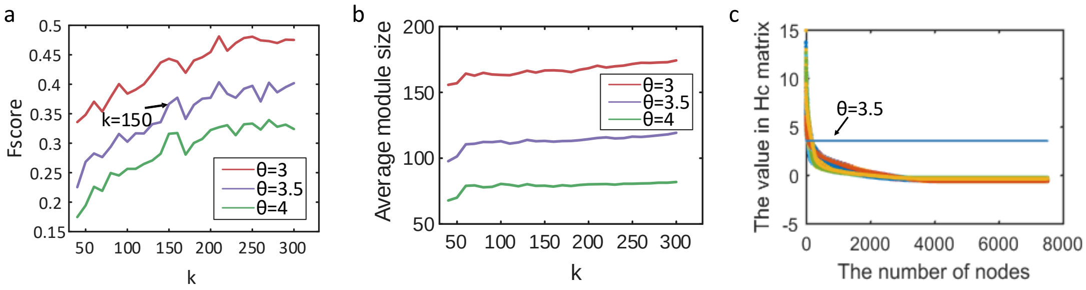

Figure S6 The impact of parameters on cancer type-specific gene co-expression networks**.** (a) The f-score values of ConMod when varying the number of candidate modules k from 40 to 300. Results on $\theta = 3, 3.5$ and 4 are given. The f-scores have no significant increase after $k = 150$. (b) The average module size w.r.t. the number of candidate modules. (c) The values with a descending order in the consensus factor matrix $\mathbf{Hc}$. We select $\theta = 3.5$ to get module members with significant high values in each column of $\mathbf{Hc}$ matrix.

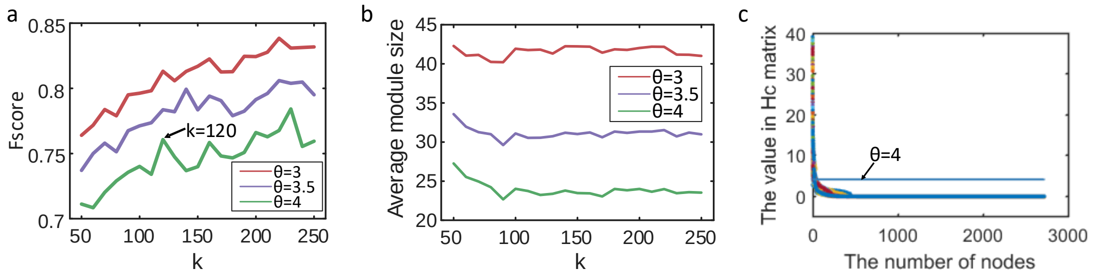

Figure S7 The impact of parameters on brain tissue-specific PPI networks**.** (a) The f-score values of ConMod when varying the number of candidate modules k from 50 to 250. Results on $\theta = 3, 3.5$ and 4 are given. The f-scores have no significant increase after $k = 120$. (b) The average module size w.r.t. the number of candidate modules. (c) The values with a descending order in the consensus factor matrix $\mathbf{Hc}$. We select $\theta = 4$ to get module members with significant high values in each column of $\mathbf{Hc}$ matrix.

References:

1. Futreal PA, Coin L, Marshall M, Down T, Hubbard T, Wooster R, Rahman N, Stratton MR: **A census of human cancer genes**. *Nature Reviews Cancer* 2004, **4**:177.
2. Kuppili Venkata S, Repana D, Nulsen J, Dressler L, Bortolomeazzi M, Tourna A, Yakovleva A, Palmieri T, Ciccarelli FD: **The Network of Cancer Genes (NCG): a comprehensive catalogue of known and candidate cancer genes from cancer sequencing screens**. *bioRxiv* 2018.
